# Systematic FTIR Spectroscopy Study of the Secondary Structure Changes in Human Serum Albumin under Various Denaturation Conditions

**DOI:** 10.3390/biom9080359

**Published:** 2019-08-12

**Authors:** Dmitrii Usoltsev, Vera Sitnikova, Andrey Kajava, Mayya Uspenskaya

**Affiliations:** 1 Department of Applied Optics, ITMO University, St.-Petersburg 197101, Russia; 2 International Research Institute of Bioengineering, ITMO University, St.-Petersburg 197101, Russia; 3 Centre de Recherche en Biologie cellulaire de Montpellier (CRBM), UMR 5237 CNRS, Universit Montpellier, CEDEX 5, 34293 Montpellier, France

**Keywords:** human serum albumin, protein denaturation, IR spectroscopy, second derivative, secondary protein structure

## Abstract

Human serum albumin (HSA) is the most abundant protein in blood plasma. HSA is involved in the transport of hormones, fatty acids, and some other compounds, maintenance of blood pH, osmotic pressure, and many other functions. Although this protein is well studied, data about its conformational changes upon different denaturation factors are fragmentary and sometimes contradictory. This is especially true for FTIR spectroscopy data interpretation. Here, the effect of various denaturing agents on the structural state of HSA by using FTIR spectroscopy in the aqueous solutions was systematically studied. Our data suggest that the second derivative deconvolution method provides the most consistent interpretation of the obtained IR spectra. The secondary structure changes of HSA were studied depending on the concentration of the denaturing agent during acid, alkaline, and thermal denaturation. In general, the denaturation of HSA in different conditions is accompanied by a decrease in α-helical conformation and an increase in random coil conformation and the intermolecular β-strands. Meantime, some variation in the conformational changes depending on the type of the denaturation agent were also observed. The increase of β-structural conformation suggests that HSA may form amyloid-like aggregates upon the denaturation.

## 1. Introduction

Protein atomic structure is usually studied by X-ray crystallography or NMR spectroscopy [1]. Although these methods provide detailed information about the native stable structures of the protein, they are limited to the proteins that can self-assemble into the high-ordered crystals or are soluble at very high concentrations. Moreover, X-ray crystallography cannot provide structural information about the protein conformational changes during its unfolding. NMR spectroscopy in solutions depends also on the solubility of the proteins and is limited by the protein molecular weight [2]. Meantime, infrared (IR) and circular dichroism (CD) spectroscopy are the classical methods applied when assessing the protein conformational changes upon its denaturation.

IR spectroscopy is widely used for studying the biological objects, especially for the conformational analysis of proteins [3,4,5,6,7,8]. So far, it is widely accepted that the protein-peptide group has nine characteristic absorption bands: amide A (~3300 cm^−1^), amide B (~3100 cm^−1^), amide I (~1650 cm^−1^), amide II (~1550 cm^−1^), amide III (~1300 cm^−1^), amide IV (~735 cm^−1^), amide V (~635 cm^−1^), amide VI (~600 cm^−1^), and amide VII (~200 cm^−1^) [8,9]. The IR spectra of protein can provide the necessary information about its secondary structure since it is characterized by the absorption bands of peptide bond vibrations -CO-NH-, whose functional groups are directly involved in the formation of a particular component of the secondary structure.

Circular dichroism is one of the most common methods used for studying the protein secondary structure, but this method is limited to the optically transparent solutions that are usually characterized by low protein concentration [10,11].

The main advantage of applying the IR spectroscopy for the analysis of protein structural characteristics is the independence of this method on the protein molecule size or the physical state of the sample. Not only the hydrated films or solid-state can be easily studied, but also the aqueous solution [12], organic solvents, detergents, micelles, and phospholipid membranes, all these states allow to study the native structure of the protein [13], as well as to assess the effect of the medium on the protein structure.

Amide I band (1600–1700 cm^−1^), which corresponds to C=O stretching vibrations, is the most sensitive part of the protein when determining the secondary structure [14]. This bond is directly related to the backbone conformations, and each type of secondary structure has different C=O stretching. Therefore, FTIR spectroscopy is a useful tool for determining the protein secondary structure, and the amide I band is widely used to quantify the secondary structure and conformational changes of proteins and polypeptides [15,16].

Two mathematical approaches are often used to separate highly overlapping components of amide I, which arise from the presence of various secondary structural elements, and these are namely the analysis of the second derivative and Fourier self-deconvolution (FSD) method [14,17,18].

The analysis of the nth derivative may be used to separate the overlapping bands. For this purpose, the second derivative is the one applied the most often. One of the main advantages of the analysis of the second derivative is that it can be performed objectively without an arbitrary choice of deconvolution parameters.

Fourier self-deconvolution is a mathematical tool for reducing bandwidth so that overlapping bands can be separated from each other. In this method, apodization is used to resolve the overlapping lines, i.e., the truncation of the interferogram, which is used to calculate the spectrum in Fourier spectrometers using the Fourier transform. During the apodization, the bandwidth is decreased artificially, i.e., the true line-component profiles are distorted for their resolution. The analysis of the second derivative, the FSD method, and curve fitting, applied in analyzing the protein spectra, gives reasonable results for the protein secondary structure [19], consistent with each other and with X-ray data [20]. Besides, the second derivative, despite the subjectivity of determining the baseline for the derivative of the spectrum [21], has enormous advantages (it separates the components of amide I without any arbitrary input factors, such as the band half-width and enhancement factor) and gives a more accurate result [22].

Quantitative analysis of the secondary structure of a protein is based on the assumption that a protein itself can be considered as a linear sum of several fundamental elements of the secondary structure. Comparison of IR spectra and high-resolution protein X-ray diffraction data can help in establishing the necessary spectrum-structure correlations. Over the past years, many correlations have been established between the IR spectra and specific protein structures [23,24]. For example, absorption bands between 1654 and 1658 cm^−1^ are expected for the proteins with α-helical structures in the aqueous solutions [25,26]. Bands between 1642, 1638, 1632, 1627, and 1624 cm^−1^ relate to the components of the β-sheet [25]. The bands located at 1688, 1680, 1672, and 1666 cm^−1^ belong to the β-turn structure [27]. The characteristic band for the random coil is in the range of 1648 ± 2 cm^−1^. For the globular protein, the correlation coefficient between the IR and X-ray structural data on the secondary structure varies as 0.90–0.99 [20,28]. However, there are some contradictions when comparing the certain frequencies with the components of the protein secondary structure. Particularly, some researchers believe that the bands related to the random coil may refer to a frequency of 1660 cm^−1^ [29], while the others state that random coil and helix structures have the same frequencies of absorption bands [30].

Therefore, it would be interesting to re-examine the consistency of the IR spectroscopy method for determining the secondary structure, especially considering the dynamics of changes in the secondary structure of the same protein under the influence of various factors. In this paper, we thoroughly analyzed the changes in the secondary structure of human serum albumin (HSA) under the action of different denaturing agents using the method of IR spectroscopy. HSA was set as the model protein since it is an important serum protein that contributes to the colloid-arterial osmotic pressure [31,32] and provides the transport and distribution of many molecules [31], metabolites [33], and drugs [34]. The other important factor for choosing this protein as the study object is the fact that albumin is one of the most studied proteins with known 3D structure [35,36]. The HSA structure has three structurally homologous α-helical domains (I–III), each subdivided into sub-domains A and B. Assembly of this domain is asymmetric. Domains I and II are perpendicular and form T-shaped assembly. The tail of subdomain IIA has hydrophobic interactions with subdomain Ia and Ib. Domain III forms Y-shaped assembly with domain II and interacts with subdomain IIB. Thus, HSA forms a heart-shaped assembly. The addition of fatty acids causes a change in the relative position of the domains, but complex formation does not change their conformational structure [37,38].

Temperature and enthalpy of denaturation of HSA, measured by DSC (differential scanning calorimetry) at different pH values and protein concentrations, was already reported by Barone et al. [39]. Structural changes in HSA in aqueous solutions at pH and thermal denaturation were investigated using vibrational spectroscopy [40,41,42,43]. Bramanti et al. [40] analyzed the quantitative determination of the secondary structure from the IR spectra of HSA in aqueous solutions both in the native and denatured states (the latter caused by heat and acid treatment), and they have also compared the data obtained by IR and CD spectroscopy. However, a more detailed study of the protein structure, particularly, the dynamics of changes in its secondary structure that occur during the protein folding/unfolding, still needs to be considered. We suggest that studying the second derivative of the protein spectra, especially in the amide I absorption band, is the best approach to analyze the dynamics of changes in particular components of the secondary structure under the influence of various factors (temperature, pH, and the concentration of a denaturing agent). This approach allows to observe the emergence of particular secondary structures and to quantify also their percentage in the denaturated structures.

## 2. Materials and Methods

Human serum albumin (HSA) was used in the form of 20% wt. aqueous solution for infusion (Microgen, Russia) without additional purification. The solutions for chemical denaturation were prepared by mixing 30 μL of 0.63M HNO_3_ (acidic) and 30 μL of 0.0012 M KOH (alkaline) solution with 30 μL of a 20% wt. ready-for-use aqueous HSA solution. The first spectrum of acidic/alkaline denaturation was recorded 30 s after mixing. The solution for thermal denaturation was a 20% wt. aqueous HSA solution, heated in a thermostat. The solution for alcohol denaturation was prepared by mixing 1 mL of a 20% wt. aqueous HSA solution with 30–210 μL of octanol-1 or isopropanol in 30 μL increments.

IR spectra of attenuated total internal reflection (ATR) of the samples were recorded in the range of 4000–600 cm^−1^ on a Bruker Tensor 37 FT-IR spectrophotometer (Bruker, Billerica, MA, USA) using an ATR accessory (diamond coated ZnSe crystal) with a spectral resolution of 2 cm^−1^ and averaging on 164 scans. The spectra of the samples were recorded with an interval of 30 s.

To obtain a protein spectrum, the solvent spectrum was subtracted iteratively until a straight baseline was obtained in the spectral region of 2000–1750 cm^−1^.

The method of separation of the second derivative of the IR spectrum was used to track the changes in the secondary structure of HSA under the influence of various factors, as described in detail earlier [20].

The protein spectra used for analysis were obtained by subtracting the spectra of an aqueous solution using Opus 7.0 software. The secondary structure was determined by decomposing the second derivative of the amide I band in the OriginPro 2015 software (OriginLab Corporation, Northampton, MA, USA), by the method described in [20,22]. All second derivatives were multiplied by −1 for easy decomposition. The control sample was a 20% wt. aqueous HSA solution.

## 3. Results

**Figure 1:**
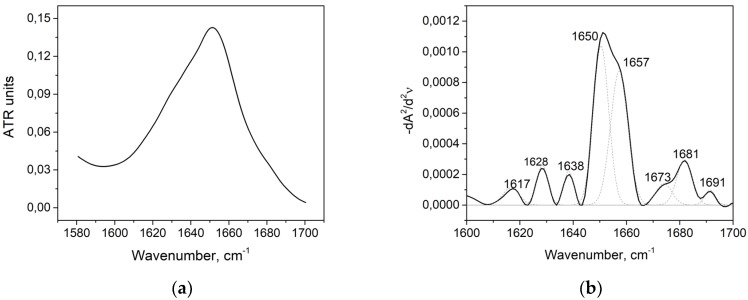
Amide I band (**a**) and its second derivative deconvolution (**b**) in the control sample. ATR: attenuated total internal reflection.

According to the previous IR spectroscopy studies of globular proteins, Amid I area of HAS (Figure 1a) and the peaks obtained from the second derivative deconvolution of this area (Figure 1b) could be correlated with the HSA secondary structure, as shown in Table 1.

Total peaks of 1650 cm^−1^ and 1657 cm^−1^ referred to 69.3% of the total area of the second derivative, which is in good agreement with the data obtained from the DSSP (Define Secondary Structure of Proteins), STRIDE (Structural Identification) algorithms for X-ray analysis (Table 2: the 1AO6 code in the Protein Data Bank) [44,45,46,47,48]. No maximum at second derivative that would correspond to a random coil defined by the range of 1640–1649 cm^−1^ was found, which is also consistent with the previous studies [49,50]. The maxima of the second derivative (1628 cm^−1^, 1638 cm^−1^, and 1691 cm^−1^) characterize the β-sheet content, referring to the study of serum albumin [50].

### 3.1. Acid Denaturation of HSA

HSA structure can be presented in the different isomeric states depending on the pH [31]: there is a negatively charged N-form (normal) in the blood at pH = 7.4; a positively charged F-form (fast) in the acidic medium at pH = 4 and E-form (extended) at pH below 3; a negatively charged B-form (basic) at pH = 8 and A-form (aged) at pH = 10 in an alkaline medium. All these isomeric states are reversible. Isoelectric points of fatted HSA and defatted has are 4.8 and 5.6, respectively [51]. Thus, F- and E-forms are expected to be present in the acidic medium. The F-form is characterized by a less compact state of the globule and a slight decrease of the α-helix content. The domain III of HSA is assumed to have a weaker structure and loses helicity at a higher value of pH = 4, while domains I and II do not lose helicity even at pH = 3.5. However, pronounced pH decrease causes the transformation of the domain II into a molten globular shape, whereas domain I does not suffer strong changes in the secondary structure [52]. The E-form is characterized by an even greater globule extension due to the repulsion of positively charged domains and an even greater decrease of helicity. Besides, an increase of β-sheet number is expected in both acidic and alkaline mediums [38,52].

**Figure 2:**
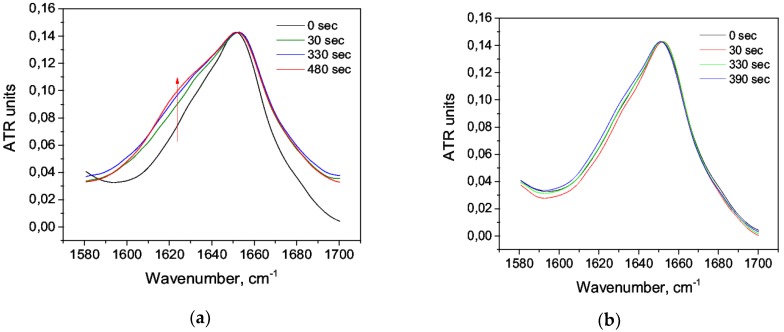
Changes in the character of the amide I band upon acid denaturation: (**a**) C = 0.63 M, (**b**) C = 1.2 mM.

The addition of nitric acid C = 0.63 M caused a strong change in the amide I band in the region 1620–1640 cm^−1^ (Figure 2) corresponding to β-sheets [53]. There were also less noticeable changes that could be studied in more detail by deconvolution of the second derivatives of the spectra (Figure 1b). At a low concentration of C = 1.2 mM, the changes were less pronounced (Figure 2b).

**Figure 3:**
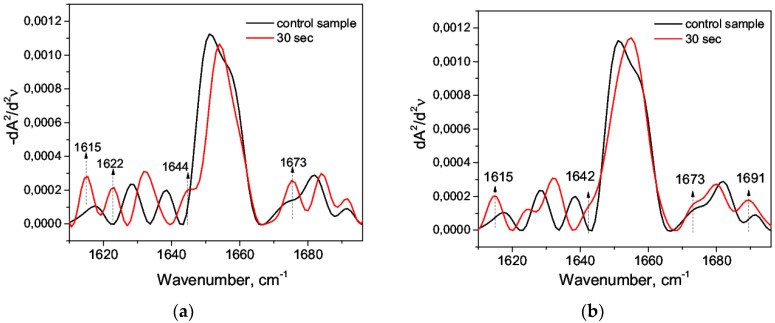
The second derivative of the amide I band: (**a**) C = 0.63 M, native form (*1*), 30 s after mixing with acid (*2*); (**b**) C = 1.2 mM, native form (*1*), 30 s after mixing with acid (*2*).

It is suggested that HSA undergoes a “molten globule” conformation with increased hydrophobicity in an acidic medium due to the globule loosening, and it retains the secondary structure to a greater degree [54,55]. The denaturing agent (nitric acid) caused the changes in the second derivatives of the amide I band. Firstly, at C = 0.63M, the deconvolution maximum 1657 cm^−1^ disappeared, and the decrease of the total area under the major maximum was obvious: this indicated a decrease of the α-helix content. It could be explained by the unfolding of domain III during acidification [54]. Secondly, new second derivative maxima appeared: 1644 cm^−1^ and 1640 cm^−1^ at C = 0.63 M and 1640 cm^−1^ at C = 1.2 mM, which corresponded to the range of random coil (Figure 3).

**Figure 4:**
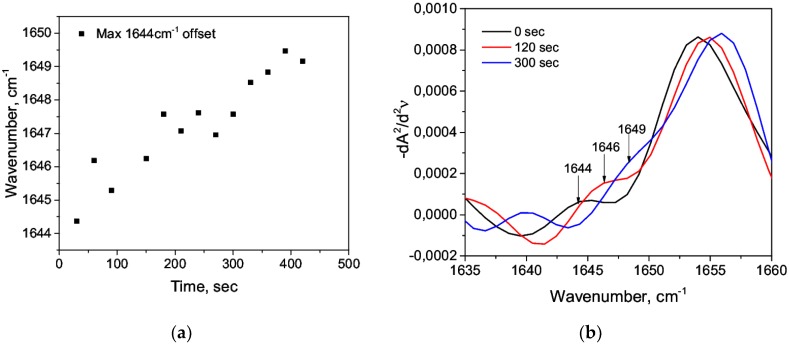
Shift (**a**) and relative increase (**b**) in the maximum of 1644 cm^−1^ to the short-wave region and its subsequent imposition on the region of 1650–1660 cm^−1^ at C = 0.63 M.

**Figure 5:**
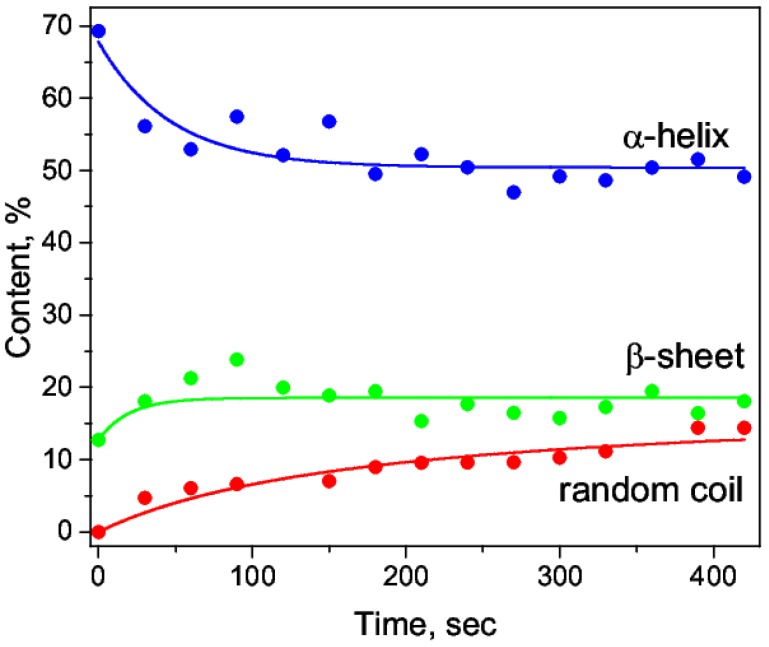
The changes in the content of α-helix, β-sheets, and the random coil structure during acid-conditioned denaturation.

During the sequential recording of the spectra (the time lag between the spectra taken was 30 s), a quantitative increase in the band at 1644 cm^−1^ and a shift to the shortwave region were observed (Figure 3 and Figure 4). Presumably, it revealed the growth of a random coil due to a decrease of α-helices. The addition of nitric acid C = 0.63 M immediately caused a reduction in α-helix by 19% due to the emerging of a random coil of 4.7% and an increase of β-sheets (Table 3). A further decrease of the α-helix and the growth of the random coil (Figure 5) were due to an increase of the maximum 1644 cm^−1^; in particular, it was due to its shift to the shortwave region (Figure 4a). When the concentration of acid was low (C = 1.2 mM), a decrease of α-helices was also present but to a lesser extent and without visible dynamics, which was also characterized by the appearance of a random coil (Table 3). Thus, we argued on the transitions α-helix → random coil and α-helix → β-sheets in the acidic medium. This was consistent with the general concepts of the E- and F-forms [52].

The primary data of the HSA secondary structure in the media characterized by different pH, obtained using the deconvolution method of the second derivative, are presented in Table 3 and Table 4. As shown in Table 4, there was a fluctuation in the α-helix content at the time intervals of 30 to 420 s. These variations might reflect time-dependent rearrangements of HSA conformation during denaturation.

There was a pronounced dynamics of changes in the secondary structure components (Figure 5). Particularly, an increase in the intermolecular β-sheets and random coil and a decrease in α-helices were observed (Table 3 and Table 4; Figure 5). The higher the concentration of the denaturing agent, the greater the change was in the proportion of these structures.

### 3.2. Alkaline Denaturation of HSA

**Figure 6:**
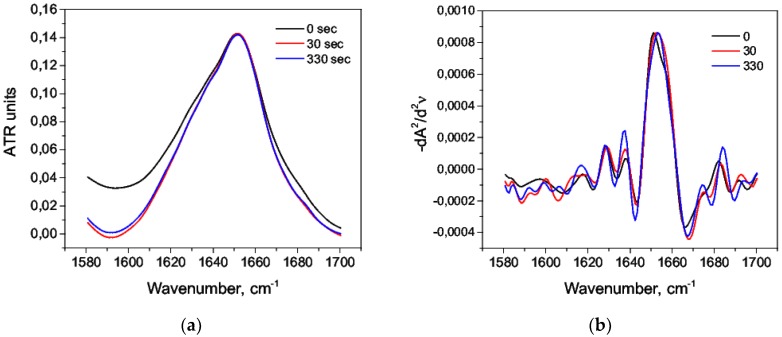
Changes in the character of the amide I band (**a**) and its second derivative (**b**) in an alkaline solution C = 1.2 mM.

Two isomers of the native form of HSA have been found in an alkaline medium: B-form (basic, at pH ~ 8) and A-form (aged, pH ~ 10) [31]. Both forms are characterized by a slight loss of helicity (by about 10%) and increased content oβ-sheets (by about 8%). At the same time, random coil conformation should not be observed [38,52,56]. The second derivative deconvolution of amide I could not distinguish such structure (Table 5, Figure 6). A decrease of the α-helix structure by an average of 10%, and an increase of β-sheets by 8% were recorded, this result corresponded to the previous studies [31,38]. Thus, in the alkaline medium, the transition α-helix → β-sheets was originally observed. This was consistent with the general concepts of the B-form [52,56].

### 3.3. Thermal Denaturation of HSA

Thermal protein denaturation can be described by the Lumry and Eyring model, which includes two steps: (1) reversible unfolding of the native protein and (2) subsequent irreversible changes that fold a new protein state, which is different from the native form and is unable to re-nature [57]. During the heating, the hydrogen bond network weakens gradually, and the buried groups become exposed to the solvent. This causes supramolecular aggregation [58]. Thus, heat treatment of the protein solution favors the formation of the protein aggregates, and the melting of the latter causes the irreversible protein unfolding. This is accompanied by a significant increase in the content of intermolecular β-sheets that are characterized by stronger hydrogen bonds [49,59]. For HSA, the aggregation begins at 56 °C, and the irreversible fibrillation starts at about 70 °C. These processes are accompanied by the loss of α-helix content [60,61]; probably, the last is partially transformed into a random coil according to the Zimm-Bragg model [62]. The melting of the α-helix in HSA is gradual and can be represented as an S-shaped curve; the α-helix content depends on the temperature according to [63].

**Figure 7:**
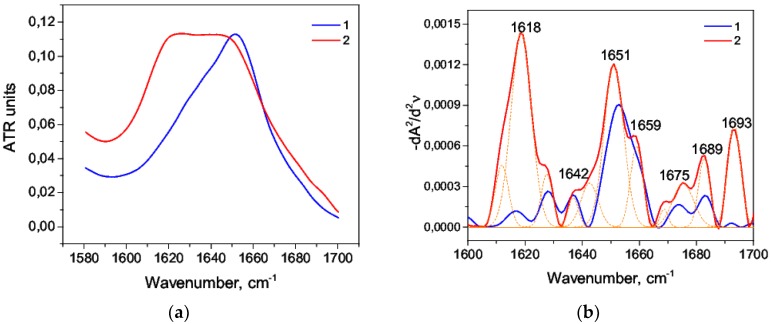
Amide I band (**a**) and the second derivative (**b**) of native (*1*) and thermally denatured (*2*) HSA.

When the protein re-naturation was stopped [64], the IR spectra in the range of the amide I band at 25 °C and 90 °C looked different (Figure 7a). The amide I band, as expected, was strongly shifted to the region of 1610–1620 cm^−1^, which corresponded to the intermolecular β sheets.

The second derivative deconvolution of amide I (Figure 7b) allowed us to distinguish two maxima: 1611 cm^−1^ and 1618 cm^−1^, and their area was 35% of the total area. Particularly, the proportion of intermolecular β-sheets increased by about 10.2 times compared with the control sample (Table 6). Besides, the total area of maxima 1651 cm^−1^ and 1659 cm^−1^ was 30%. This result indicated that the content of α-helix decreased by about 2.3 times. The maximum 1642 cm^−1^ indicated the presence of a random coil (Table 3). The second derivative deconvolution allowed to evaluate all the changes even visually (Figure 7b).

**Figure 8:**
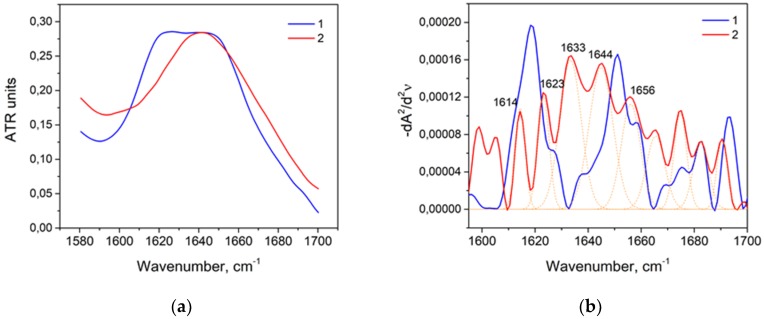
Thermally denatured amide I band (*1*) and thermally denatured HSA in the alkaline medium (*2*): primary data (**a**) and their second derivatives (**b**).

The temperature of 90 °C in combination with an alkaline solution (1M KOH) of HSA (20% *w*/*w*) for 1 min caused a significant reduction of the α-helix [53] and an increment of β-sheets (Table 7). Unlike alkaline denaturation without heating, random coil content is expected [65]. In contrast to the neutral medium, protein aggregates did not form in an alkaline medium, and the content of intermolecular β-sheets was low (Table 7). In the second derivative of the amide I band, there were no significant peaks in the region of 1610–1620 cm^−1^, but other pronounced peaks could be identified: 1645 cm^−1^, corresponding to the random coil; 1623 cm^−1^ and 1633 cm^−1^, corresponding to β-sheets, which exceeded their content in the native state almost threefold, and the α-helix content was about 80% lower compared to the native state (Figure 8).

**Figure 9:**
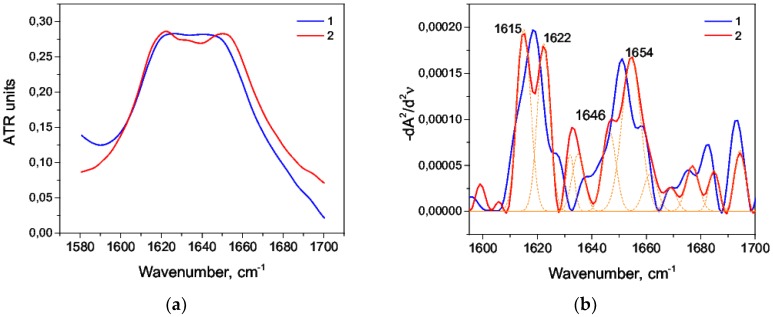
Thermally denatured amide I band (*1*) and thermally denatured HAS at 90 °C in an acidic medium (*2*): primary data (**a**) and their second derivatives (**b**).

The exposing of the acidic solution (1M HNO_3_) of HSA (20% *w*/*w*) at a temperature of 90 °C for 1 min caused the reduction of the α-helix content and a significant formation of intermolecular β-sheets (Figure 9) [31,66]. Alongside with that, the peak 1646 cm^−1^ exceeded that observed at thermal denaturation in a neutral medium by 1.6 times. This indicated that acid isomerization of HSA occurred to a greater extent due to the temperature. Therefore, this result referred to a combination of acid (at t = 25 °C) and thermal (in a neutral medium) denaturation.

### 3.4. The Effect of Alcohol on the HSA Secondary Structure

Alcohols are known to have two main effects on a protein solution: destabilization of the tertiary structure and stabilization of the helical secondary structure at low alcohol concentrations [67]. It has been reported earlier that β-sheets are expected to increase due to a decrease of α-helicity at high concentrations of alkyl alcohols. There is a conformational switch from α-helix to β-sheet [67,68]. Moreover, these effects increase with an increase in the length of an alcohol hydrocarbon chain [38]. This is confirmed by the fact that albumin undergoes aggregation in ethanol solutions and that it is represented as the extended globule state with same or higher helicity as the native form [31,67].

It is known that the hydrophobicity of non-polar side chains plays a significant role in the stability of the protein globule in the aqueous solutions. Its importance is comparable to that of the hydrogen bonding and van der Waals interaction of polar groups [69]. Non-polar side chains will tend to occupy a position within the globule in a polar solvent like water is. Burial of more than 80% of these non-polar side chains allows the globular protein to stabilize the natural compact structure [69]. However, when the hydrophilic-lipophilic balance of the solvent changes, the globular structure can unfold and so interact with the solvent efficiently in terms of energy. The free energy of transfer for peptide groups and a non-polar leucine side chain from water to various solvents has been obtained earlier [69]. The authors report that, for example, the free energy of transfer for peptide groups and a non-polar leucine side chain from water to urea is negative. This means that peptide groups and leucine side chains will aim to interact with the urea, and so the protein will unfold. When using alcohols as a solvent, the pattern is different: for example, the free energy of transfer for peptide groups from water to ethanol will be +1400 cal mol^−1^, but –1800 cal mol^−1^ for leucine side chain; therefore, the tertiary structure will be destructed, and the globule will expand. However, after the unfolding of the protein molecule, the α- helix refolding is often observed. By folding into α-helices, polar peptide groups may be buried in the interior of the helix, and non-polar side chain will be in contact with ethanol outside the helix [69]. Thus, the helicity will not decrease. A similar pattern will be observed for other aliphatic alcohols. Besides, the aliphatic alcohols have been previously reported to disturb the tertiary structure of HSA, but they stabilize its secondary structure [38].

**Figure 10:**
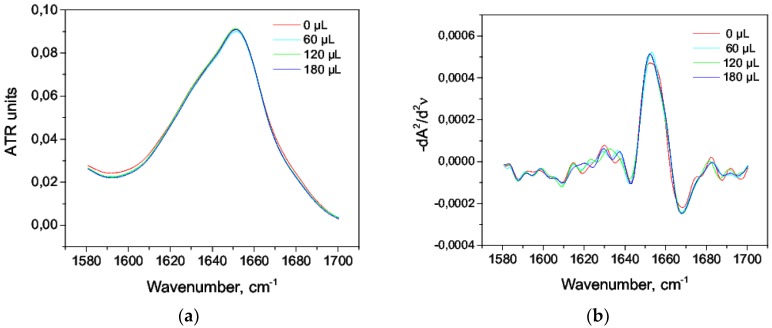
Amide I bands in the aqueous solution of octanol-1 (**a**) and their second derivatives (**b**).

**Figure 11:**
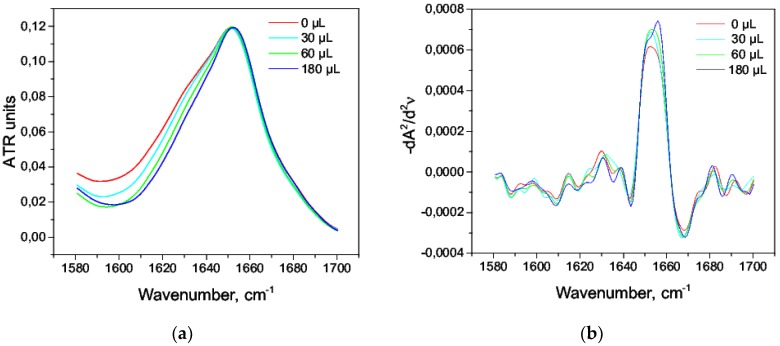
Amide I bands in the aqueous solution of isopropanol (**a**) and their second derivatives (**b**).

We observed the effects of isopropanol and octanol-1 in the range of 3–17% (*v*/*v*) on HSA solution 20% (*w*/*w*). Within the studied range of concentrations, both isopropanol and octanol-1 did not reduce the helicity, did not change the content of β-sheets, and did not cause the growth of intermolecular β sheets; also, there was a slight increase in helicity by 1–2% (Table 8 and Table 9). The amide I band practically did not change over the whole range of concentrations of octanol-1 and was similar to native state (Figure 10). The same pattern was observed for second derivatives (Figure 10b). There were slight visible changes in the amide I band when using isopropanol (Figure 11a); however, it had practically no effect on the second derivative (Figure 11b). This could be explained by a stronger stabilizing effect of octanol-1 compared with isopropanol.

**Figure 12:**
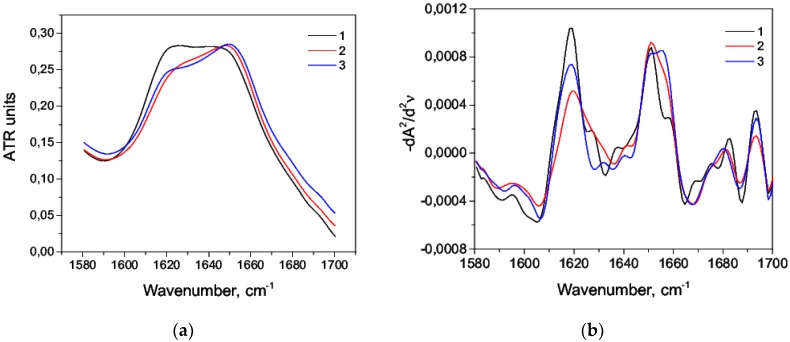
Amide I bands of HSA (**a**) and their second derivatives (**b**) at 90 °C in the pure aqueous solution (*1*), in the aqueous solution of octanol-1 (*2*), and the aqueous solution of isopropanol (*3*).

The stabilizing effect of alcohol was also observed during thermal denaturation of HSA. At 90 °C, amide I bands differed greatly in water-alcohol solution comparing to pure water solution, mainly in the region corresponding to the intermolecular β-sheets (Figure 12a). This was also noticeable for the second derivatives (Figure 12b). The addition of 2.9% (*v*/*v*) octanol-1 in the aqueous albumin solution preserved the helicity by 13% more compared to the pure water solution and reduced the intermolecular β-sheets content by 8.5% (Table 10). Similar results were observed for 5.7% (*v*/*v*) isopropyl alcohol but to a lesser extent. This again indicated a greater stabilizing ability of octanol-1 compared with isopropanol.

## 4. Conclusions

**Figure A1:**
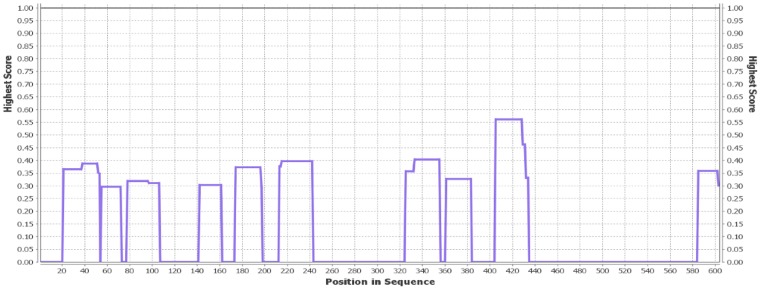
Prediction of amyloidogenic regions (denoted by blue contours) in the unfolded HSA by using ArchCandy program.

The IR spectroscopic method had been applied to study HSA conformational changes upon denaturation. The effect of various denaturation agents on the secondary structure of human serum albumin was studied by the method of the second derivative. The dynamics of changes in the individual components of the secondary structure varied depending on the type and concentration of the denaturation agent. At high concentrations of nitric acid, the random coil conformation increased noticeably and continued to grow over time, while at lower acid concentrations, such changes were not so pronounced. The percentage of β-sheets increased almost twofold at high concentrations and by 1.5 times at low concentrations of nitric acid. Therefore, we could track the conformational transition from the initial α-helical conformation to both random coil conformation and β-structures. With alkaline denaturation, the main trend was the decrease of α-helicity and increase of the β-structural content. During thermal denaturation, a significant increase in the proportion of intermolecular β-sheets was observed (approximately 10.2 times as compared with the control sample), accompanied by a decrease in the proportion of α-helix (approximately 2.3 times). In contrast to the neutral medium, we did not observe protein aggregates in an alkaline medium, as evidenced by a small fraction of intermolecular β-structures. However, the proportion of β-sheets still exceeded this value in the native form almost threefold, and the α-helix content was approximately 80% lower than that observed for the native state. The combined effect of temperature and the acidic medium led to melting of the α-helical structure and the formation of intermolecular β -structures in high numbers. All these data suggest that upon the denaturation, HSA may form β-structure rich in amyloid-like aggregates. Indeed, our analysis of the HSA amino acid sequence by using ArchCandy program [70] revealed that a significant part of this protein had an amyloidogenic region, which has a potential to form cross-β-amyloids when unfolded (Figure A1). On the contrary, alcohols have a stabilizing effect on albumin and practically do not change its secondary structure.

Finally, our analysis of the IR spectroscopy data, using the method of the second derivative, evidenced that this method was suitable not only for detecting the small changes in the secondary structure of the protein but also for their quantification.

## Figures and Tables

**Table 1:** The correlation between the HSA’s (human serum albumin) secondary structure and the second derivative of amide.

Wavenumber, cm^−1^	Range, cm^−1^	Secondary Structure
1618	1610–1619	Intermolecular β-sheet
1628, 1638, 1691	1620–16139, 1689–1695	β-sheet
1650, 1657	1650–1660	α-helix
1673, 1681	1660–1689	β-turn

**Table 2:** The HSA secondary structure.

Data Source	α-helix, %	β-sheet, %	β-turn, %	Random Coil, %	Irregular Structure, %
FTIR ^1^	69.30	12.72	14.57	0	0
DSSP ^2^ [37,38,39]					
Chain A Chain B	68.38 69.23	0 0	8.89 7.18	0 0	12.99 12.99
STRIDE ^3^ [37,40,41]					
Chain A Chain B	69.06 69.06	0 0	15.70 14.52	0 0	12.48 12.82
X-ray analysis [37,42]	68.8	7.3	2.1		21.8

^1^ Fourier-transform infrared spectroscopy; ^2^ Define Secondary Structure of Proteins; ^3^ Structural Identification.

**Table 3:** Deconvolution (%) in 20% solution of HSA (*w*/*w*) + 0.63 M HNO_3_.

Time, sec	α-Helix, %	β-Sheet, %	β-Turn, %	Random Coil, %	Intermolecular β-Sheet, %
0	69 ± 2	13 ± 2	14.5 ± 2	-	3 ± 2
30	56 ± 2	17 ± 2	16 ± 2	5 ± 2	6 ± 2
60	53 ± 2	18 ± 2	15 ± 2	6 ± 2	8 ± 2
90	57 ± 2	19 ± 2	12 ± 2	6 ± 2	5 ± 2
120	52 ± 2	18 ± 2	18 ± 2	5 ± 2	6 ± 2
150	57 ± 2	16 ± 2	17 ± 2	7 ± 2	3 ± 2
180	50 ± 2	17 ± 2	19 ± 2	8 ± 2	6 ± 2
210	52 ± 2	18 ± 2	14 ± 2	9 ± 2	6 ± 2
240	50 ± 2	21 ± 2	16 ± 2	8 ± 2	4 ± 2
270	47 ± 2	19 ± 2	17 ± 2	10 ± 2	7 ± 2
300	49 ± 2	20 ± 2	14 ± 2	10 ± 2	6 ± 2
330	49 ± 2	19 ± 2	17 ± 2	10 ± 2	5 ± 2
360	50 ± 2	18 ± 2	14 ± 2	13 ± 2	5 ± 2
390	51 ± 2	18 ± 2	11 ± 2	13 ± 2	6 ± 2
420	49 ± 2	19 ± 2	12 ± 2	14 ± 2	6 ± 2

^1^ Note. Here and in Table nos. 4—9, the data are presented as M ± *m* (where applicable), where M and m are the parameter value and its deviation, respectively.

**Table 4:** Deconvolution (%) in 20% solution of HSA (*w*/*w*) + 1.2 mM HNO_3_.

Time, sec	α-Helix, %	β-Sheet, %	β-Turn, %	Random Coil, %	Intermolecular β-Sheet, %
0	69 ± 2	13 ± 2	15 ± 2	-	3 ± 2
30	62 ± 2	18 ± 2	15 ± 2	1.6 ± 2	5 ± 2
60	55 ± 2	21 ± 2	14 ± 2	5 ± 2	4 ± 2
90	55 ± 2	24 ± 2	11 ± 2	6 ± 2	4 ± 2
120	55 ± 2	22 ± 2	13 ± 2	5 ± 2	5 ± 2
150	59 ± 2	19 ± 2	12 ± 2	4 ± 2	5 ± 2
180	64 ± 2	19 ± 2	8 ± 2	3 ± 2	5 ± 2
210	69 ± 2	15 ± 2	11 ± 2	2 ± 2	4 ± 2
240	58 ± 2	24 ± 2	12 ± 2	2 ± 2	5 ± 2
270	69 ± 2	17 ± 2	9 ± 2	0 ± 2	5 ± 2
300	64 ± 2	16 ± 2	13 ± 2	2 ± 2	5 ± 2
330	67 ± 2	17 ± 2	11 ± 2	2 ± 2	5 ± 2
360	64 ± 2	19 ± 2	12 ± 2	0	5 ± 2
390	67 ± 2	16 ± 2	12 ± 2	0	5 ± 2
420	64 ± 2	18 ± 2	15 ± 2	0	6 ± 2

**Table 5:** The deconvolution of HSA (%) during the peak amide I in a solution 20% HSA + 1.2 mM KOH.

Time, sec	α-Helix, %	β-Sheet, %	β-Turn, %	Random coil, %	Intermolecular β-Sheet, %
0	69 ± 2	14.5 ± 2	13 ± 2	0	3 ± 2
30	59 ± 2	13 ± 2	20 ± 2	0	8 ± 2
60	63 ± 2	12 ± 2	20 ± 2	0	5 ± 2
90	58 ± 2	15 ± 2	20 ± 2	0	6 ± 2
120	61 ± 2	13 ± 2	18 ± 2	0	8.5 ± 2
150	61 ± 2	14 ± 2	20 ± 2	0	7 ± 2
180	60 ± 2	13 ± 2	19 ± 2	0	7 ± 2
210	62 ± 2	14 ± 2	19 ± 2	0	5 ± 2
240	58 ± 2	12 ± 2	22 ± 2	0	8 ± 2
270	61 ± 2	13 ± 2	20 ± 2	0	6 ± 2
300	59 ± 2	13 ± 2	20 ± 2	0	7 ± 2
330	60 ± 2	13 ± 2	21 ± 2	0	6 ± 2
360	60 ± 2	12 ± 2	19 ± 2	0	8 ± 2
390	60 ± 2	14 ± 2	19 ± 2	0	7 ± 2
420	62 ± 2	13 ± 2	18 ± 2	0	7 ± 2

**Table 6:** The secondary structure of HSA (%) during thermal denaturation.

Temperature, °C	α-Helix, %	β-Sheet, %	β-Turn, %	Random Coil, %	Intermolecular β-Sheet, %
25	69 ± 2	13 ± 2	14.6 ± 2	0	3 ± 2
90	30 ± 2	16 ± 2	13 ± 2	5.5 ± 2	35 ± 2

**Table 7:** Thermal denaturation (%) of HSA in various media at 90 °C.

Medium	α-Helix, %	β-Sheet, %	β-Turn, %	Random Coil, %	Intermolecular β-Sheet, %
Neutral	30 ± 2	16 ± 2	13 ± 2	5.5 ± 2	35 ± 2
Alkaline (1M KOH)	13 ± 2	34 ± 2	23.6 ± 2	22 ± 2	6.6 ± 2
Acidic (1M HNO_3_)	23.7 ± 2	15 ± 2	10.5 ± 2	9 ± 2	37.7 ± 2

**Table 8:** The second derivative deconvolution (%) of the amide I band of HSA 20% (*w*/*w*), octanol-1 added.

Alcohol, mL	α-Helix, %	β-Sheet, %	β-Turn, %	Random Coil, %	Intermolecular β-Sheet, %
0	61 ± 2	21 ± 2	14 ± 2	0	4 ± 2
30	60 ± 2	20 ± 2	16 ± 2	0	4 ± 2
60	62 ± 2	19 ± 2	11 ± 2	0	4.6 ± 2
90	62 ± 2	18 ± 2	14 ± 2	0	4 ± 2
120	63 ± 2	17.5 ± 2	13 ± 2	0	4 ± 2
150	64 ± 2	17 ± 2	13 ± 2	0	5.5 ± 2
180	63 ± 2	20.6 ± 2	14 ± 2	0	2.5 ± 2
210	62 ± 2	21 ± 2	13.5 ± 2	0	2.7 ± 2

**Table 9:** The second derivative deconvolution (%) of the amide I band of HSA 20% (*w*/*w*), isopropanol added.

Alcohol, mL	α-Helix, %	β-Sheet, %	β-Turn, %	Random Coil, %	Intermolecular β-Sheet, %
0	61 ± 2	21 ± 2	14 ± 2	0	4 ± 2
30	63.6 ± 2	19 ± 2	13.6 ± 2	0	4 ± 2
60	63 ± 2	20 ± 2	12 ± 2	0	4 ± 2
90	61.6 ± 2	20 ± 2	14.5 ± 2	0	4 ± 2
120	60 ± 2	21.5 ± 2	14.4 ± 2	0	4 ± 2
150	63 ± 2	20 ± 2	14 ± 2	0	2.6 ± 2
180	62 ± 2	22.4 ± 2	12.5 ± 2	0	3 ± 2
210	64 ± 2	18 ± 2	14.5 ± 2	0	3 ± 2

**Table 10:** Thermal denaturation of HSA (%) in alcohol solution

Medium	α-Helix, %	β-Sheet, %	β-Turn, %	Random Coil, %	Intermolecular β-Sheet, %
Alcohol-free	30 ± 2	16 ± 2	13 ± 2	5.5 ± 2	35 ± 2
Octanol-1 (3%)	43 ± 2	15 ± 2	13 ± 2	2.7 ± 2	26 ± 2
Isopropanol (6%)	37 ± 2	13.5 ± 2	10 ± 2	8 ± 2	32 ± 2
